# Effects of aerobic exercise on cognition, sleep, and mood in healthy adults: a systematic review and meta-analysis of randomized controlled trials

**DOI:** 10.3389/fnhum.2026.1808755

**Published:** 2026-05-29

**Authors:** Sitong Wang, Jiayun Chen, Chuang Gao, Yuchen Zhou, Zhen Wei, Zhiqiang Liang

**Affiliations:** 1 Faculty of Sport Science, Ningbo University, Ningbo, Zhejiang, China; 2 School of Exercise and Health, Nanjing Sport Institute, Nanjing, China

**Keywords:** acute exercise, aerobic exercise, cognitive function, emotional regulation, long-term exercise, meta-analysis, sleep quality

## Abstract

This study evaluated the effects of aerobic exercise on cognitive function, sleep, and emotional states in healthy adults through a systematic review and meta-analysis. Literature published within the past 5 years was retrieved from five databases: PubMed, Web of Science, Elsevier ScienceDirect, CNKI, and VIP. A total of 19 randomized controlled trials were included. The results showed that acute aerobic exercise significantly enhanced positive mood (SMD = 1.56) and reduced negative mood (SMD = −0.46), but did not yield significant improvements in cognitive performance. In contrast, long-term aerobic exercise effectively alleviated negative mood (SMD = −0.58), whereas its effects on positive mood, cognitive function, and sleep quality were not statistically significant. These findings suggest that acute exercise may serve as an effective strategy for rapid emotional regulation, while regular long-term exercise contributes beneficially to emotional well-being. However, the potential benefits of aerobic exercise on cognition and sleep warrant further investigation through more standardized and high-quality studies.

**Systematic review registration:**

https://www.crd.york.ac.uk/PROSPERO/view/CRD420261383739, CRD420261383739.

## 1 Introduction

With the accelerating pace of modern life and increasing work-related stress, healthy adults commonly experience subclinical issues such as cognitive fatigue, deteriorated sleep quality, and emotional fluctuations (Huang et al., 2019). Although these symptoms do not meet clinical diagnostic criteria, they significantly impair individuals’ quality of life, work productivity, and long-term health. In this context, physical activity has gained widespread attention as a safe, low-cost, and easily implementable non-pharmacological intervention (Warburton and Bredin, 2017). A growing body of evidence suggests that aerobic exercise may exert multidimensional beneficial effects on cognitive function, sleep regulation, and emotional states (Kandola et al., 2018). In particular, acute aerobic exercise, a single bout of exercise, has been shown to enhance attention and positive mood in the short term, while long-term aerobic exercise, regular training over several weeks or more, may improve memory function and sleep quality through mechanisms involving neuroplasticity (Basso and Suzuki, 2017).

Epidemiological and cross-sectional studies have provided strong support for the health benefits of physical activity. Multiple prospective cohort studies have demonstrated that higher levels of physical activity are significantly associated with a lower risk of cognitive decline, even after adjusting for age, education level, vascular risk factors, and genotype, with this association remaining robust. Neuroimaging evidence further indicates that individuals who exercise regularly exhibit greater gray matter density and better white matter integrity in key brain regions such as the prefrontal cortex and hippocampus, suggesting potential neuroprotective effects (Sofi et al., 2011). However, despite the consistency of observational findings, evidence from randomized controlled trials (RCTs) remains highly heterogeneous, making it difficult to draw definitive conclusions (Tseng et al., 2013).

Existing systematic reviews indicate that multimodal interventions combining aerobic and resistance training components may have relatively greater potential for improving cognitive function, particularly executive function, in older adults. A meta-analysis including 25 RCTs supports this view, but also highlights that the number of studies on such combined interventions remains limited and their long-term effects are still unclear (Zhang et al., 2023). Furthermore, variability in exercise parameters may be a key factor contributing to inconsistent findings. Studies suggest that moderate-intensity aerobic exercise, with moderate acute duration, regular frequency, and sufficient total intervention duration, is closely associated with significant improvements in overall cognitive function (Erickson et al., 2011). Although resistance training is often implemented at lower frequencies, it can still effectively enhance executive function when performed with moderate duration. Notably, high-intensity multimodal exercise interventions with good continuity and regularity have also shown positive effects on memory (Northey et al., 2018). However, it remains unclear whether there is a difference in cognitive outcomes between moderate-intensity constant training and progressively increasing intensity regimens, warranting further investigation (Kelly et al., 2014).

In addition, current studies lack standardization in the measurement tools used for cognition, sleep, and mood, limiting the comparability of results (Lampit et al., 2014). There is also inconsistency across studies in defining acute versus long-term exercise, which undermines the integration of evidence. Particularly among healthy adults, there is a lack of systematic reviews and meta-analyses that simultaneously compare the multidimensional effects of acute and long-term aerobic exercise on cognition, sleep, and mood (Zhang et al., 2023).

Therefore, this study aims to systematically retrieve and conduct a meta-analysis of existing high-quality RCT evidence to comprehensively evaluate the effects of aerobic exercise on cognitive function, sleep quality, and emotional states in healthy adults. Subgroup analyses will be performed based on intervention type, exercise parameters, and assessment tools, with the goal of enhancing the comparability, interpretability, and practical applicability of the findings.

## 2 Methods

### 2.1 Search strategy

This study systematically searched the following databases: PubMed, Web of Science, Elsevier ScienceDirect, China National Knowledge Infrastructure (CNKI), and VIP Information Network (VIP). The search period was limited to studies published from January 1, 2020, to September 1, 2025, to include recent high-quality RCTs with rigorous study designs. Restricting the search to the past 5 years ensures the inclusion of studies employing standardized intervention protocols and contemporary assessment tools, thereby enhancing the clinical relevance and methodological consistency of the findings. The detailed search strategies and keyword combinations for each database was presented in Table 1.

**Table 1:** Search keywords and strategies in the included databases.

Database	Search terms
Pub Med	(“Cognition”[MeSH Terms] OR cognition[tiab] OR “cognitive function”[tiab]) AND (“Emotions”[MeSH Terms] OR emotion[tiab] OR mood[tiab] OR feeling[tiab]) AND (“Exercise”[MeSH Terms] OR exercise[tiab] OR aerobic exercise[tiab] OR physical activity[tiab]) AND (“Sleep”[MeSH Terms] OR sleep[tiab] OR insomnia[tiab] OR sleep quality[tiab]) AND (2020:2025[pdat])
Web of science	TS = ((cognition OR cognitive function) AND (emotion OR mood OR feeling) AND (exercise OR aerobic exercise OR physical activity) AND (sleep OR insomnia OR sleep quality)) AND PY = (2020–2025)
Elsevier ScienceDirect	TITLE-ABS-KEY ((cognition OR “cognitive function”) AND (emotion OR mood OR feeling) AND (exercise OR “aerobic exercise” OR “physical activity”) AND (sleep OR insomnia OR “sleep quality”)) AND PUBYEAR > 2019 AND PUBYEAR < 2026
CNKI	Topic = (cognition OR cognitive function) AND (emotion OR mood OR feeling) AND (exercise OR aerobic exercise OR physical activity) AND (sleep OR insomnia OR sleep quality) AND Year of Publication > = 2020 AND <= 2025
VIP	Title or Keywords = (cognition OR cognitive function) AND (emotion OR mood OR feeling) AND (exercise OR aerobic exercise OR physical activity) AND (sleep OR insomnia OR sleep quality) AND Publication Year > = 2020 AND <= 2025

MeSH, medical subject headings, tiab, title/abstract, TS, topic search, PY, publication year.

### 2.2 Inclusion and exclusion criteria

Studies were included if they met all of the following criteria:

(1) Study design: RCTs, including parallel-group or crossover designs, regardless of blinding status.
(2) Participants: 18 ≤ Adults aged <80 years, without diagnosed neurological disorders, psychiatric conditions, severe chronic diseases, or cognitive impairments.
(3) Intervention: Aerobic exercise interventions such as running, brisk walking, cycling, swimming, or aerobics, with clearly defined exercise type, intensity, frequency, and duration.
(4) Control condition: Control groups engaged in no exercise, usual daily activity, placebo-like low-intensity activities, or non-aerobic interventions.
(5) Outcome measures: At least one of the following outcomes was reported with quantitative data: cognitive function, sleep quality, emotional state.
(6) Language and publication period: Full-text articles published in English or Chinese between January 1, 2020, and April 1, 2025.
(7) Data availability: Studies reported means and standard deviations, t-values, *F*-values, *p*-values, effect sizes, or provided raw data sufficient for calculating effect sizes.
(8) Study type: both peer-reviewed journal articles and accessible academic theses or dissertations were included to minimize publication bias.

Studies were excluded if they met any of the following criteria:

(1) Study design: Non-RCT study designs.
(2) Participants were children, adolescents, older adults, or clinical populations.
(3) Interventions did not involve aerobic exercise.
(4) No control group was included, or the control group received an intervention essentially similar to aerobic exercise, exclusion in such cases depended on the availability of a true control condition.
(5) No cognitive, sleep, or emotional outcomes were reported, or outcomes were described only qualitatively without standardized, quantitative assessments.
(6) Insufficient or missing data that prevented data extraction or effect size calculation.

### 2.3 Data extraction

Two independent reviewers extracted data from all included studies. The following information was collected: article title, author, publication year, study design, total sample size, number of participants in intervention and control groups, participant characteristics, details of the intervention, cognitive assessment tools, sleep assessment tools, emotional assessment tools, and key outcome data. Data were entered into a standardized Excel spreadsheet and cross-checked by a third reviewer for consistency. All extracted data were used for subsequent meta-analysis. The data extraction form was presented in Table 2.

**Table 2:** Characteristics of included studies.

No.	Author (year)	Sample size intervention group control group	Intervention protocol	Cognitive measures	Sleep measures	Emotional measures
1	Wu and Zhou (2024)	44 22 22	Exercise type: treadmill running Frequency: single acute Duration per acute: 30 min	—	—	TAS, TAI, PANAS, POMS
2	Cui (2021)	47 24 23	Exercise type: cycle ergometer Frequency: single acute Duration per acute: 30 min	Customized dual-task experimental paradigm or simply customized dual-task paradigm	—	
3	Liao (2023)	92 72 20	Exercise type: cycle ergometer Frequency: single acute Duration per acute: 30 min	Study-recognition task paradigm or learning-recognition paradigm	—	PANAS
4	Qin (2023)	60 45 15	Exercise Type: cycle ergometer Frequency: single acute Duration per acute: 30 min	Facial emotion recognition task paradigm or commonly shortened to facial emotion recognition task	—	WLELS
5	Xiao (2020)	66 30 36	Exercise type: indoor bike Frequency: single acute Duration per acute: 20 min	—	—	Emotion induction experiment or mood induction procedure (MIP)
6	Zhou (2024a,b)	48 24 24	Exercise type: cycle ergometer Frequency: single acute Duration per acute: 30 min	—	—	IAPS
7	Nouchi et al. (2020)	59 29 30	Exercise type: Push-ups Frequency: single acute Duration per acute: 30 min	rST, ST	—	POMS-SF2
8	Yildirim et al. (2024)	75 60 15	Exercise type: treadmill running Frequency: single acute Duration per acute: 30 min	PASAT	—	—
9	Luo (2022)	41 21 20	Exercise type: stationary bike Frequency: 3 times per week Duration per acute: 35 min Total duration: 4 weeks	—	PSQI	GAD-7 PHQ-9
10	Molina-Hidalgo et al. (2025)	83 64 19	Exercise type: high-intensity interval training Frequency: 2 times per week Duration per acute: 40–65 min Total duration: 10 weeks	—	—	BDI-II /PSS /POMS /PANAS/SHS
11	Ni et al. (2024)	80 40 40	Exercise type: high-intensity interval training Frequency: 5 times per week Duration per acute: 30 min Total duration: 4 weeks	N-back	PSQI	HAMA,HAMD
12	Wang and Boros (2020)	58 42 16	Exercise type: treadmill running and cycle ergometer Frequency: 4 times per week Duration per acute: 50 min	—	PSQI	STAI,SDS
13	Zhou et al. (2024a,b)	50 25 25	Exercise Type: Baduanjin Frequency: 3 times per week Duration per acute: 30 min Intensity: low to moderate intensity Total duration: 3 months	MMSE, MOCA	—	
14	Xu et al. (2025)	121 61 60	Exercise type: treadmill running Total duration: 12 weeks	MMSE	HAMD-17	
15	Cunha et al. (2025)	149 75 74	Exercise type: Baduanjin Frequency: 3 times per week Duration per acute: 60 min Total duration: 12 weeks	MOCA	PSQI	BAI,PHQ-9
16	Kargaran et al. (2021)	24 16 8	Exercise type: treadmill running Frequency: 3 times per week Duration per acute: 20 min Total duration: 8 weeks	MMSE	PSQI	SF-36
17	Wang and Boros (2020)	26 14 12	Exercise type: walking Frequency: 7 times per week Total duration: 4 weeks	—	PSQI	—
18	You et al. (2023)	50 35 15	Exercise type: running Frequency: 3 times per week Total duration: 12 weeks	Stroop	—	—
19	Liu et al. (2025)	78 47 31	Exercise type: Baduanjin Frequency: 5 times per week Duration per session: 12 min Total duration: 10 weeks	SHMS, visual memory test	—	—

### 2.4 Risk of bias assessment

The methodological quality of included studies was assessed using the Cochrane Collaboration’s - Risk of Bias 2 (RoB 2) tool for randomized trials, as recommended in the *Cochrane Handbook for Systematic Reviews of Interventions* (Higgins et al., 2022). Five domains were evaluated:

(1) Bias arising from the randomization process.
(2) Bias due to deviations from intended interventions.
(3) Bias due to missing outcome data.
(4) Bias in measurement of the outcome.
(5) Bias in selection of the reported result.

Each domain was rated as low risk, some concerns, or high risk of bias. Assessments were conducted independently by two reviewers, with disagreements resolved through discussion or consultation with a third reviewer.

### 2.5 Statistical analysis

The Standardized Mean Difference (SMD) with 95% confidence intervals (CI) was used as the primary effect size. To account for potential dependence among effect sizes, a multilevel random-effects model was employed, with parameter estimation conducted using restricted maximum likelihood (REML). Outcomes measured with different instruments were pooled. When the same scale was used across studies, mean difference (MD) was calculated. A random-effects model was applied to aggregate data, accommodating inter-study heterogeneity. Heterogeneity was assessed using the chi-square test and the *I*^2^ statistic. *I*^2^ < 50% was interpreted as low heterogeneity, while *I*^2^ ≥ 50% indicated moderate to high heterogeneity. The overall effect was tested for statistical significance using the *Z*-test, with *p* < 0.05 considered statistically significant. If 10 or more studies were included, sensitivity analyses and subgroup analyses (by intervention duration, frequency, etc.) were conducted. Publication bias was visually inspected using funnel plots. All analyses were performed using Review Manager 5.4.1 (The Cochrane Collaboration).

## 3 Results

### 3.1 Study selection

**Figure 1:**
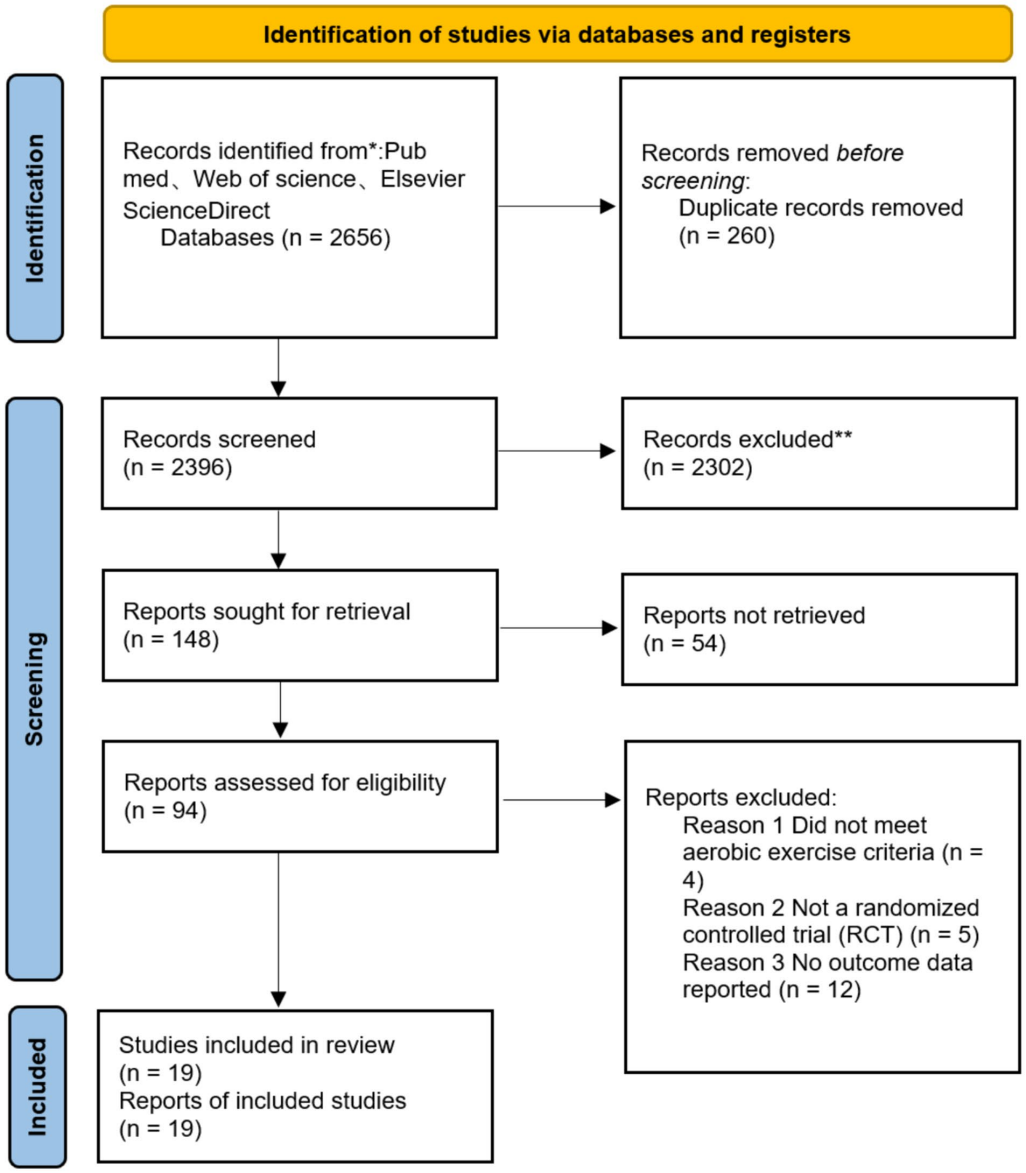
PRISMA flow diagram of study selection.

The initial database search yielded 2,656 records. After removing duplicates, 2,396 studies remained. A total of 2,302 records were excluded based on title and abstract screening, leaving 94 studies for full-text assessment. Of these, 61 were excluded for the following reasons: incomplete data (*n* = 54), non-aerobic intervention (*n* = 4), non-RCT design (*n* = 5), or lack of relevant outcome measures (*n* = 12). Ultimately, 19 RCTs were included in the meta-analysis (Figure 1).

### 3.2 Risk of bias assessment

**Figure 2:**
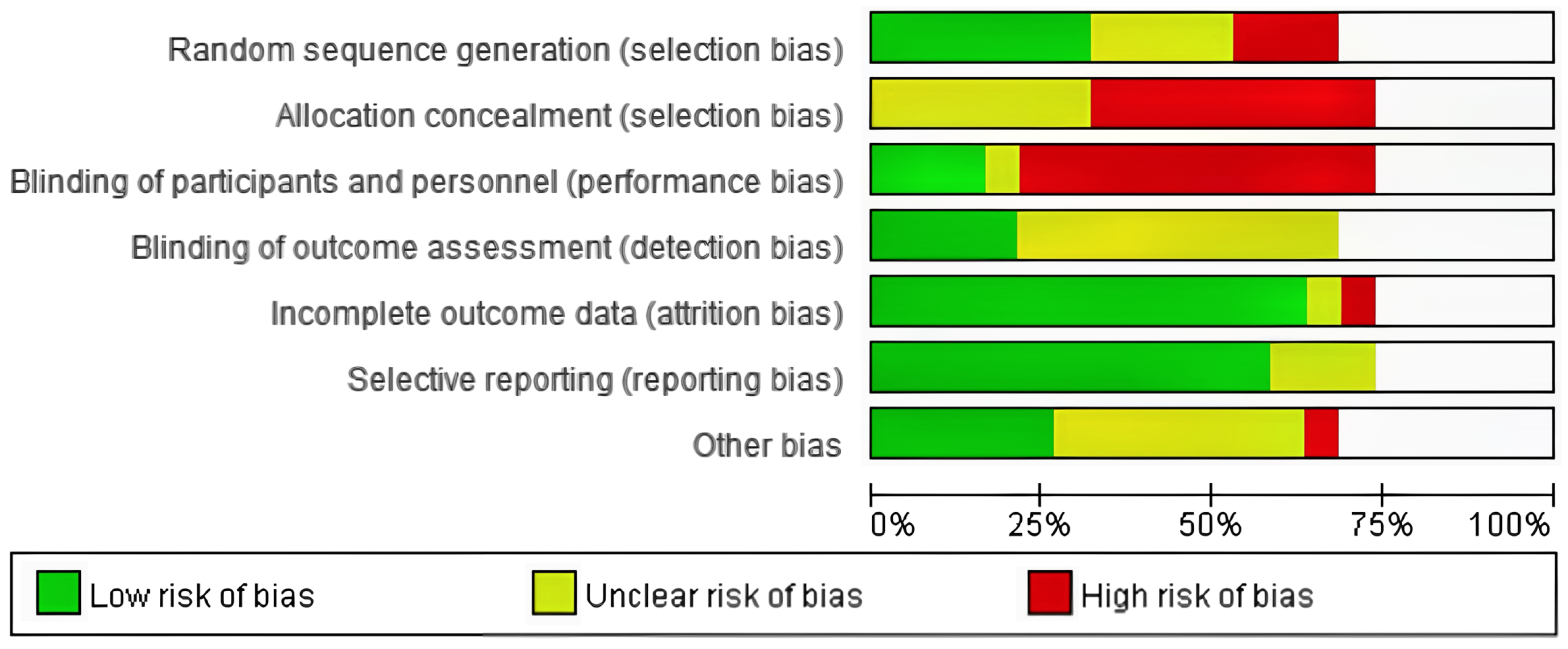
Risk of bias summary for included studies.

The risk of bias for all included randomized controlled trials was assessed using the Cochrane Collaboration’s RoB 2.0 tool. Two reviewers independently conducted the evaluation across the following seven domains. Each domain was judged as having low risk, uncertain risk, or high risk of bias. Domain-level assessment (N = 19 RCTs):

(1) Randomization process: 6 studies (32%) at low risk, 5 (26%) with some concerns, 8 (42%) at high risk;
(2) Deviations from intended interventions (i.e., blinding of participants/personnel): 3 (16%) low risk, 10 (53%) some concerns, 6 (32%) high risk;
(3) Missing outcome data: 10 (53%) low risk, 4 (21%) some concerns, 5 (26%) high risk; Measurement of the outcome (blinding of outcome assessors): 5 (26%) low risk, 9 (47%) some concerns, 5 (26%) high risk;
(4) Selection of the reported result (selective reporting): 8 (42%) low risk, 7 (37%) some concerns, 4 (21%) high risk;
(5) Other biases: 5 (26%) low risk, 8 (42%) some concerns, 6 (32%) high risk. The final results are presented in a summary risk of bias graph (Figure 2).

### 3.3 Overall analysis

#### 3.3.1 Characteristics of included studies

A total of 19 RCTs, published between 2020 and 2025, were included in this review, with a combined sample size of 1,251 healthy adults—746 participants in the intervention groups and 505 in the control groups. The age of participants ranged from 18 to 70 years, with most studies recruiting sedentary university students or middle-aged adults.

The primary forms of aerobic exercise interventions included brisk walking (Wang and Boros, 2020), running (Wu and Zhou, 2024; Yildirim et al., 2024; Zhou, 2024a; Kargaran et al., 2021; You et al., 2023), and cycling (Wang and Boros, 2020; Cui, 2021; Liao, 2023; Qin, 2023; Xiao, 2020; Zhou, 2024b; Luo, 2022; Nouchi et al., 2020), Push-ups (Nouchi et al., 2020), and high-intensity interval training (Molina-Hidalgo et al., 2025; Ni et al., 2024), Baduanjin (Cunha et al., 2025; Liu et al., 2025; Xu et al., 2025), predominantly performed at moderate intensity. Of the 19 studies, eight investigated acute aerobic exercise (Wu and Zhou, 2024; Yildirim et al., 2024; Cui, 2021; Liao, 2023; Qin, 2023; Xiao, 2020; Zhou, 2024b; Nouchi et al., 2020), with single acute lasting 20–30 min and immediate post-exercise effects assessed. The remaining 11 studies examined long-term interventions (Zhang et al., 2023; Wang and Boros, 2020; Zhou, 2024a; Kargaran et al., 2021; You et al., 2023; Cui, 2021; Luo, 2022; Molina-Hidalgo et al., 2025; Ni et al., 2024; Xu et al., 2025), with durations ranging from 4 to 12 week, conducted 3–5 times per week, and acute lengths varying from 12 to 60 min.

Cognitive function was primarily assessed using standardized tools such as the Montreal Cognitive Assessment (MoCA) (Zhou, 2024a; Cunha et al., 2025), Stroop Color-Word Test (You et al., 2023), and the Sydney Holistic Memory Scale (SHMS) (Liu et al., 2025). Emotional states were evaluated using the Profile of Mood States (POMS) (Wu and Zhou, 2024; Nouchi et al., 2020; Molina-Hidalgo et al., 2025), Patient Health Questionnaire-9 (PHQ-9) (Luo, 2022), and Positive and Negative Affect Schedule (PANAS) (Wu and Zhou, 2024; Liao, 2023; Molina-Hidalgo et al., 2025). Sleep quality was measured predominantly using the Pittsburgh Sleep Quality Index (PSQI) (Wang and Boros, 2020; Kargaran et al., 2021; Luo, 2022; Nouchi et al., 2020; Ni et al., 2024; Cunha et al., 2025).

Detailed characteristics of the included studies are presented in Table 2.

#### 3.3.2 Effects of acute aerobic exercise on positive mood

**Figure 3:**
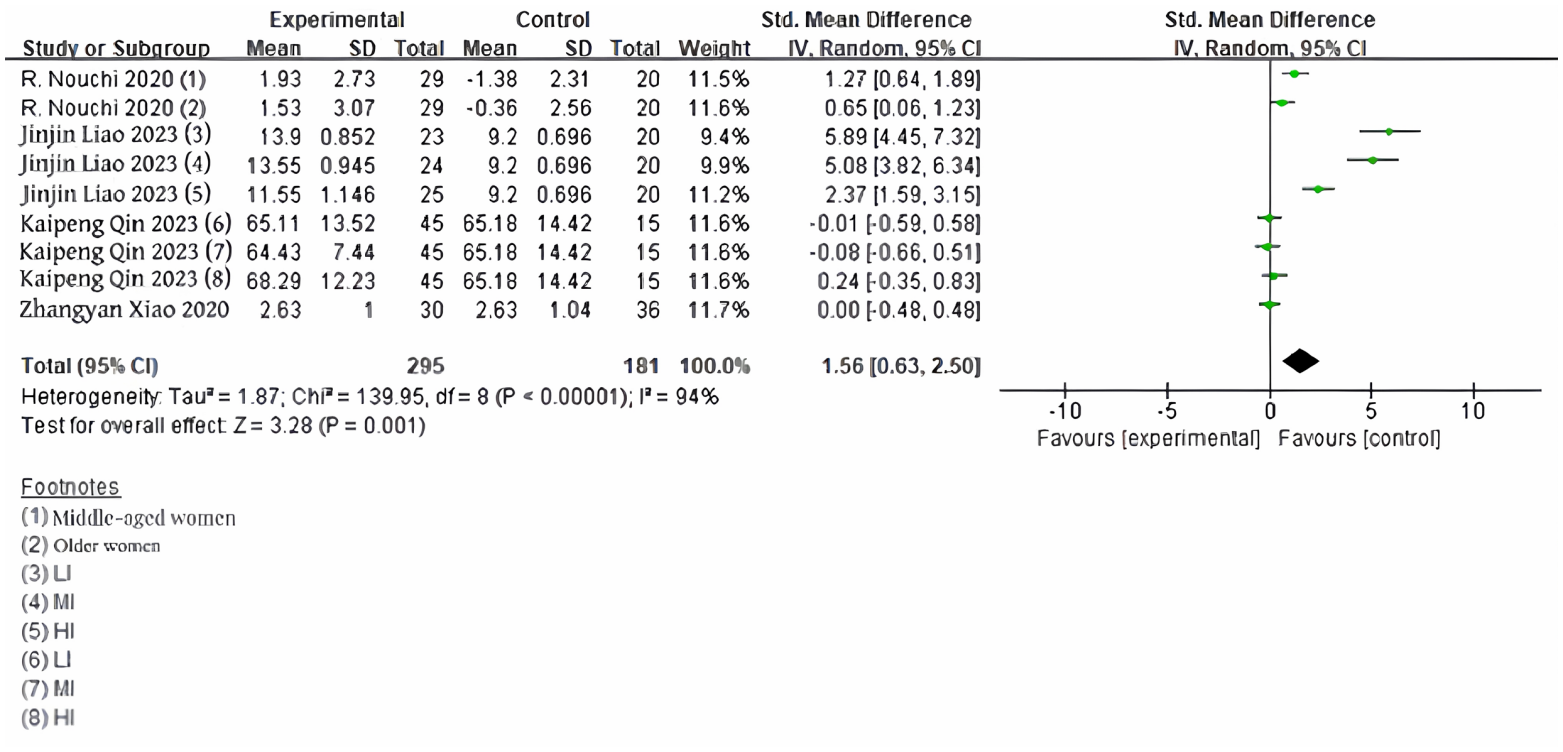
Forest plot showing the effect of acute aerobic exercise on positive mood in healthy adults.

Four studies were included to evaluate the immediate effects of a single acute of aerobic exercise on positive mood in healthy adults (Liao, 2023; Qin, 2023; Xiao, 2020; Nouchi et al., 2020). Substantial heterogeneity was observed across studies (*I^2^* = 94%, *p* < 0.00001) (Figure 3). A random-effects model was used to pool effect sizes, the pooled results showed that acute aerobic exercise significantly improved positive mood (SMD = 1.56, 95% CI [0.63, 2.50], *Z* = 3.28, *p* = 0.001).

#### 3.3.3 Effects of acute aerobic exercise on negative mood

**Figure 4:**
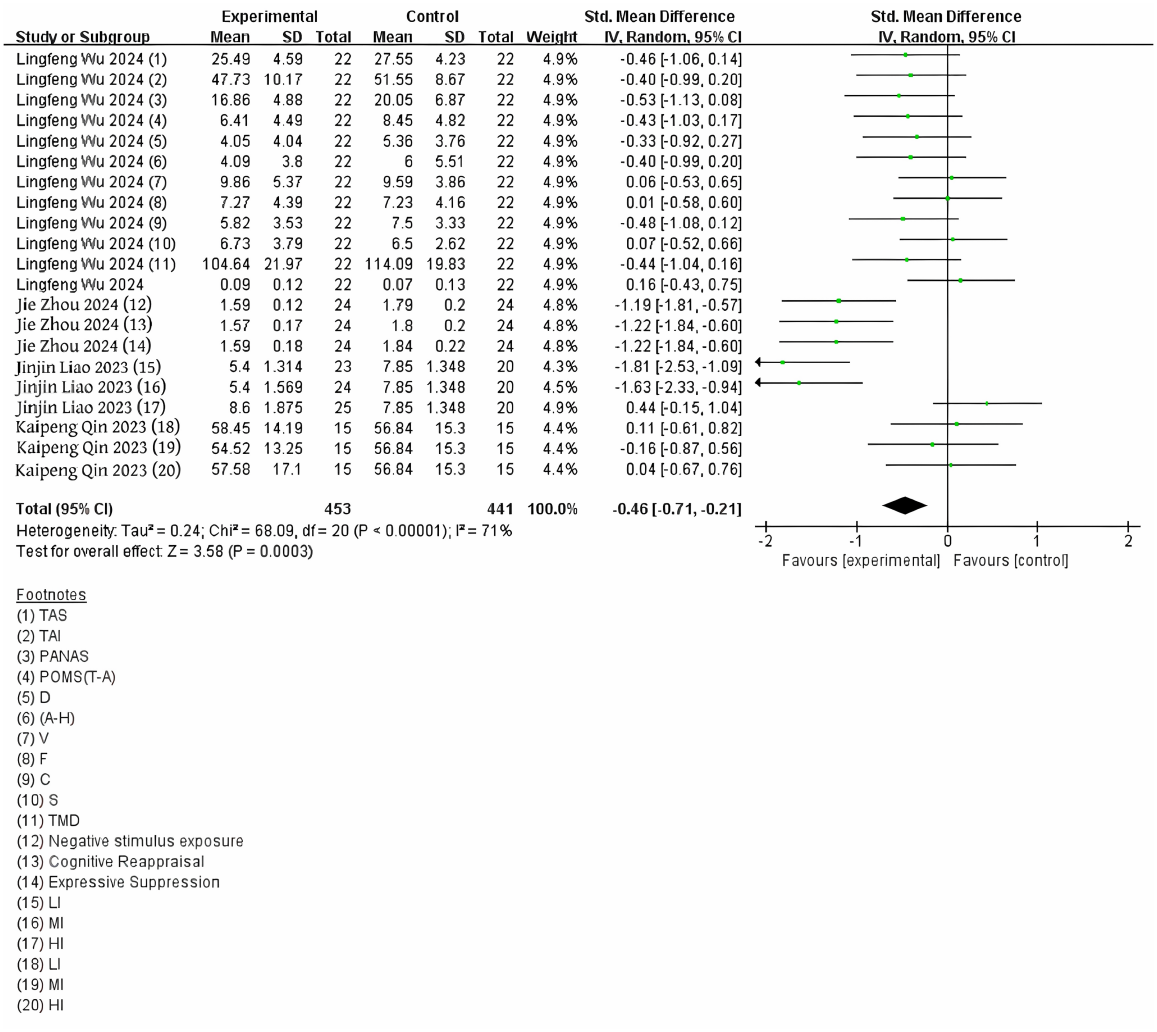
Forest plot showing the effect of acute aerobic exercise on negative mood in healthy adults.

Four studies were included to assess the impact of acute aerobic exercise on negative mood in healthy adults (Wu and Zhou, 2024; Liao, 2023; Qin, 2023; Zhou, 2024b). Moderate to high heterogeneity was observed across studies (*I*^2^ = 71%, *p* < 0.001) (Figure 4). A random-effects model was employed to pool effect sizes, the pooled results indicated that aerobic exercise significantly reduced negative mood (SMD = −0.46, 95% Cl [−0.71, −0.21], *Z* = 3.58, *p* = 0.0003).

#### 3.3.4 Effects of acute aerobic exercise on cognitive function

**Figure 5:**
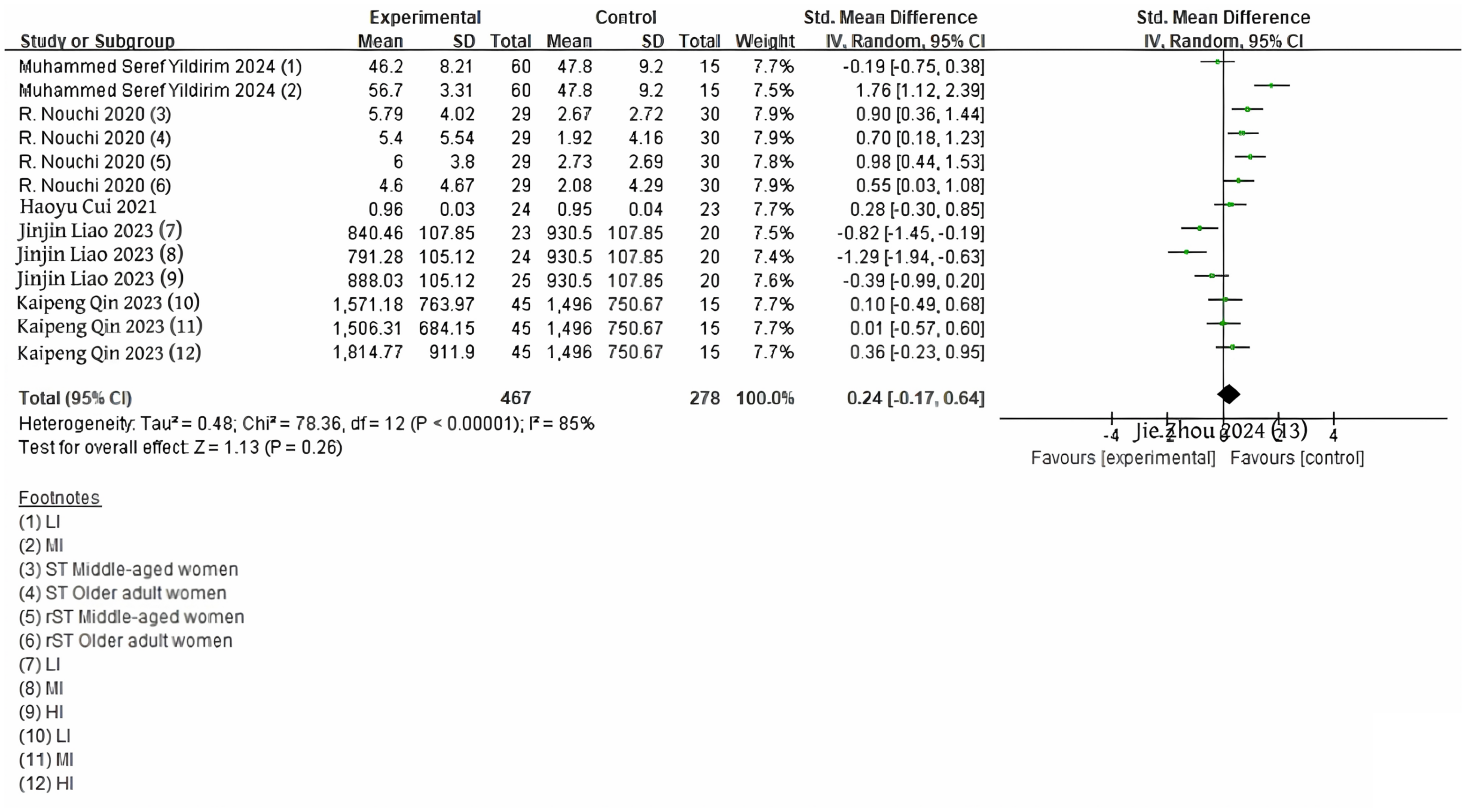
Forest plot showing the effect of acute aerobic exercise on cognitive function in healthy adults.

Five studies were included to evaluate the effects of acute aerobic exercise on cognitive function in healthy adults (Yildirim et al., 2024; Cui, 2021; Qin, 2023; Nouchi et al., 2020). Substantial heterogeneity was observed across studies (*I^2^* = 85%, *p* < 0.001) (Figure 5). A random-effects model was used to pool effect sizes, the pooled analysis showed that aerobic exercise had a non-significant effect on cognitive function (SMD = 0.24, 95% Cl [−0.17, 0.64], *Z* = 1.13, *p* = 0.26).

### 3.4 Effects of long-term aerobic exercise on mood, cognition, and sleep

#### 3.4.1 Effects of long-term aerobic exercise on positive mood

This section included two RCTs evaluating the effects of long-term aerobic exercise on positive mood in healthy adults (Kargaran et al., 2021; Xiao, 2020). The intervention durations ranged from 8 to 12 weeks, with an exercise frequency of 3–5 sessions per week at 60%–75% of maximum heart rate. Regarding measurement tools, studies utilized different scales (SF-36 and PANAS). The meta-analysis revealed no statistically significant effect of long-term aerobic exercise on positive mood compared to control conditions (SMD = −0.20, 95% CI [−0.62, 0.22], *p* = 0.35). Both individual studies reported non-significant changes, with effect sizes ranging from −0.25 to −0.05. These findings suggest that while long-term exercise may not significantly enhance positive mood in healthy adults, it does not exert negative effects either.

#### 3.4.2 Effects of long-term aerobic exercise on negative mood

**Figure 6:**
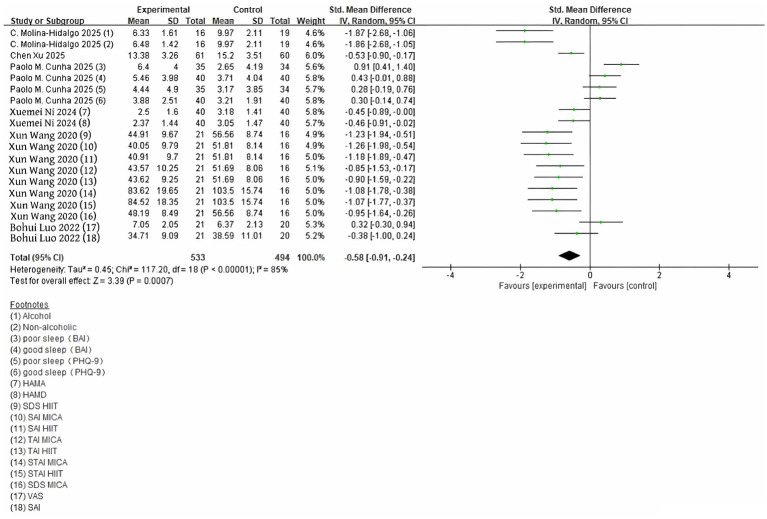
Forest plot showing the effect of long-term aerobic exercise on negative mood in healthy adults.

Six studies were included to assess the impact of long-term aerobic exercise on negative mood in healthy adults (Wang and Boros, 2020; Luo, 2022; Nouchi et al., 2020; Molina-Hidalgo et al., 2025; Ni et al., 2024; Cunha et al., 2025; Xu et al., 2025). Substantial heterogeneity was observed across studies (*I^2^* = 85%, *p* < 0.001) (Figure 6). A random-effects model was used to pool effect sizes, the pooled analysis revealed that long-term aerobic exercise significantly reduced negative mood (SMD = −0.58, 95% CI [−0.91, −0.24], *Z* = 3.39, *p* = 0.0007).

#### 3.4.3 Effects of long-term aerobic exercise on cognitive function

**Figure 7:**
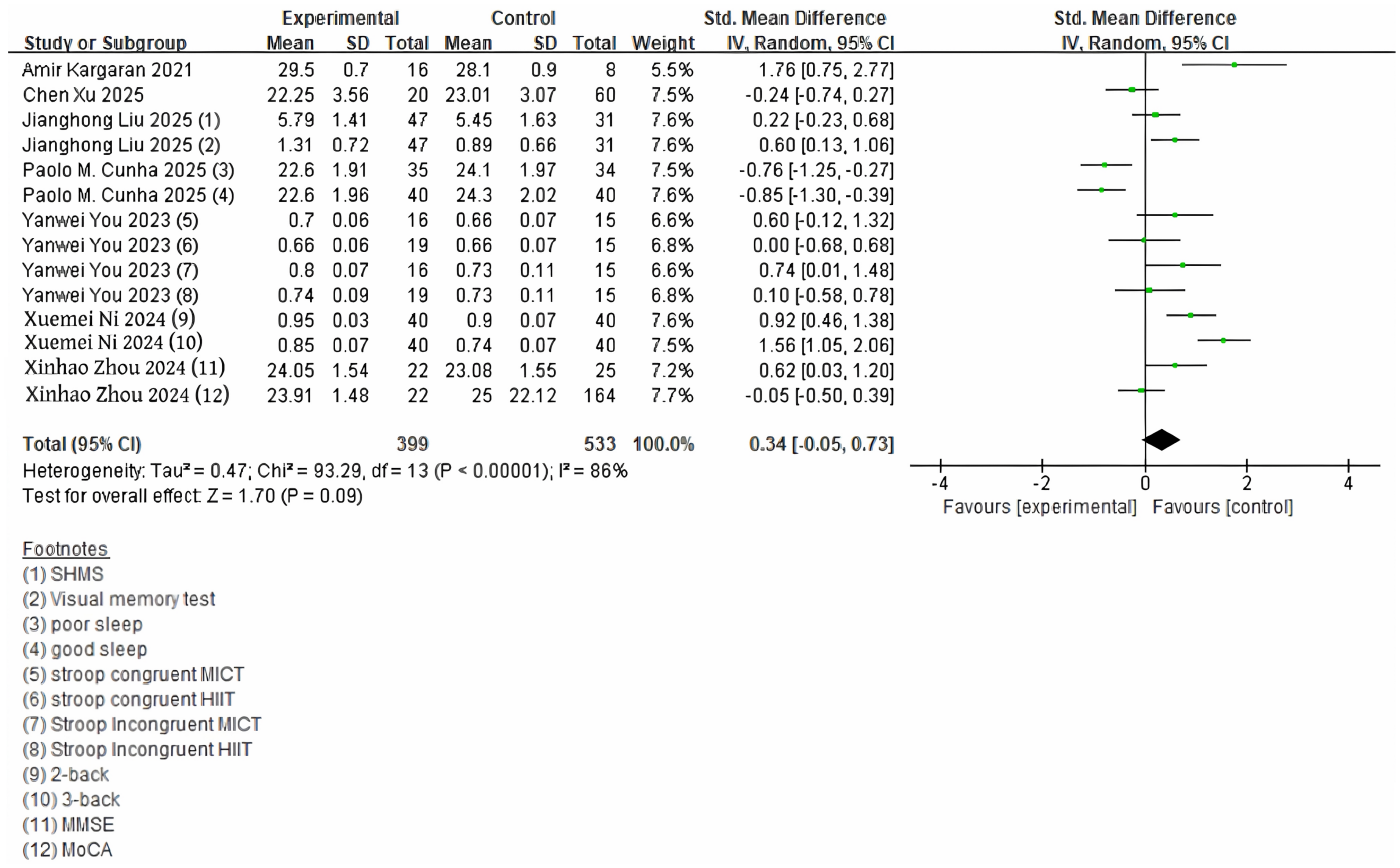
Forest plot showing the effect of long-term aerobic exercise on cognitive performance in healthy adults.

Seven studies were included to evaluate the effects of long-term aerobic exercise on cognitive function in healthy adults (Zhou, 2024a; Kargaran et al., 2021; You et al., 2023; Molina-Hidalgo et al., 2025; Ni et al., 2024; Cunha et al., 2025; Xu et al., 2025). Substantial heterogeneity was observed across studies (*I*^2^ = 86%, *p* < 0.001) (Figure 7). A random-effects model was used to pool effect sizes, the pooled analysis showed that the exercise group performed better than the control group in cognitive outcomes, but the difference was not statistically significant (SMD = 0.34, 95% CI [−0.05, 0.73], *Z* = 1.70, *p* = 0.09).

#### 3.4.4 Effects of long-term aerobic exercise on sleep quality

**Figure 8:**
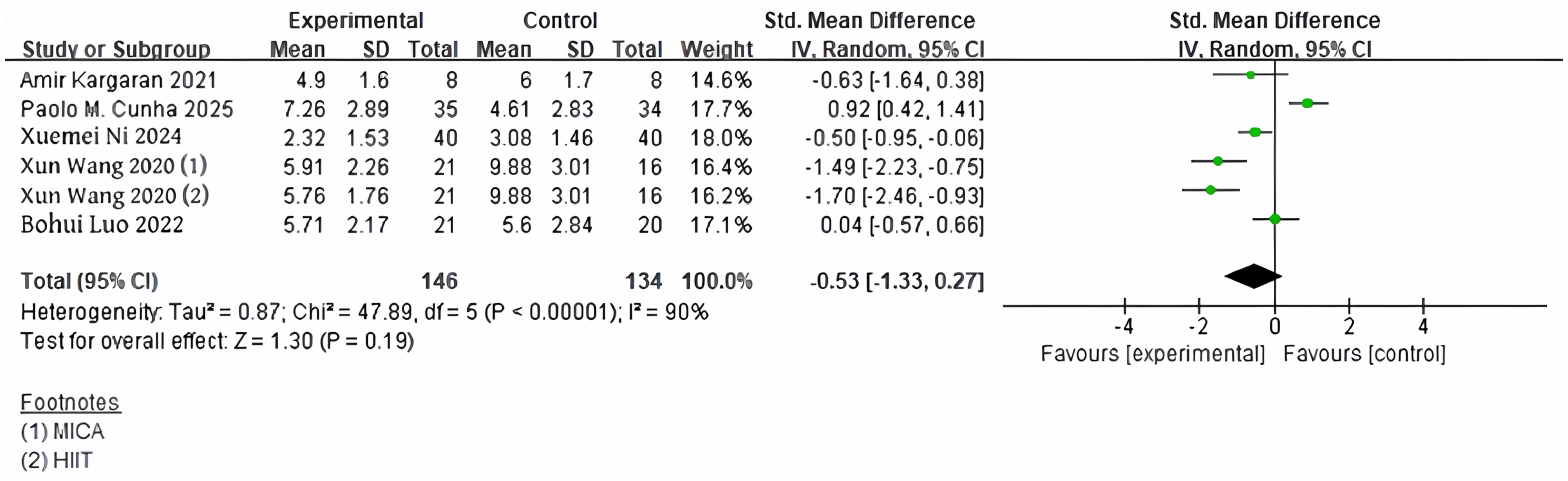
Forest plot showing the effect of long-term aerobic exercise on sleep quality in healthy adults.

Five studies were included to evaluate the effects of long-term aerobic exercise on sleep quality in healthy adults (Wang and Boros, 2020; Kargaran et al., 2021; Luo, 2022; Nouchi et al., 2020; Ni et al., 2024; Cunha et al., 2025). There was very high heterogeneity across studies (*I^2^* = 90%, *p* < 0.001) (Figure 8). A random-effects model was used to pool effect sizes, the pooled results showed a potential improvement in sleep quality. However, the difference was not statistically significant (SMD = −0.53, 95% CI [−1.33, 0.27], *Z* = 1.30, *p* = 0.19).

### 3.5 Heterogeneity and sensitivity analysis

#### 3.5.1 Acute aerobic exercise and positive mood

To assess the influence of individual studies on the pooled result, we conducted a leave-one-out sensitivity analysis by sequentially excluding each study and re-performing the meta-analysis. The results showed that the SMD ranged from 0.33 to 2.42 after removing each study, indicating a certain degree of stability in the overall findings. Notably, when the studies by Liao (2023); Nouchi et al. (2020) were removed, the pooled SMD dropped to 0.04 [(95% CI: −0.24, 0.31), *p* = 0.80], and the effect was no longer statistically significant. In all other exclusion scenarios, the effect sizes remained statistically significant (*p* < 0.05), and the 95% CI did not cross zero.

Additionally, heterogeneity fluctuated across the sensitivity analyses (*I*^2^ ranging from 66% to 96%), confirming substantial between-study heterogeneity. However, no single study altered the direction of the overall effect (Table 3).

**Table 3:** Characteristics of included studies.

Author(s)	Outcome variable	Measurement tool	Effect size (95% CI)	Notes
Wu and Zhou (2024)	Negative affect	TAS	−0.46 [−1.06, 0.14]	Single-acute intervention
		TAI	−0.40 [−0.99, 0.20]	
		PANAS	−0.53 [−1.13, 0.08]	
		POMS T-A	−0.43 [−1.03, 0.17]	
		POMS D	−0.33 [−0.92, 0.27]	
		POMS A-H	−0.40 [−0.99, 0.20]	
		POMS V	0.06 [−0.53, 0.65]	
		POMS F	0.01 [−0.58, 0.60]	
		POMS C	−0.48 [−1.08, 0.12]	
		POMS S	0.07 [−0.52, 0.66]	
		POMS TMD	−0.44 [−1.04, 0.16]	
Cui (2021)	Cognitive function	Customized dual-task paradigm	0.28 [−0.30, 0.85]	Single-acute intervention
Liao (2023)	Cognitive function	Learning-recognition task paradigm LI	−0.82 [−1.45, −0.19]	Single-acute intervention
		Learning-recognition task paradigm MI	−1.29 [−1.94, −0.63]	
		Learning-recognition task paradigm HI	−0.39 [−0.99, 0.20]	
	Positive affect	PANAS LI	5.89 [4.45, 7.32]	Single-acute intervention
		PANAS MI	5.08 [3.82, 6.34]	
		PANAS HI	2.37 [1.59, 3.15]	
	Negative affect	PANAS LI	−1.81 [−2.53, −1.09]	Single-acute intervention
		PANAS MI	−1.63 [−2.33, −0.94]	
		PANAS HI	0.44 [−0.15, 1.04]	
Qin (2023)	Cognitive function	Facial emotion recognition task paradigm LI	0.10 [−0.49, 0.68]	Single-acute intervention
	Cognitive function	Facial emotion recognition task paradigm MI	0.01 [−0.57, 0.60]	
	Cognitive function	Facial emotion recognition task paradigm HI	0.36 [−0.23, 0.95]	
	Positive affect	WLELS LI	−0.01 [−0.59, 0.58]	
	Positive affect	WLELS MI	−0.08 [−0.66, 0.51]	
	Positive affect	WLELS HI	0.24 [−0.35, 0.83]	
	Negative affect	WLELS LI	0.11 [−0.61, 0.82]	
	Negative affect	WLELS MI	−0.16 [−0.87, 0.56]	
	Negative affect	WLELS HI	0.04 [−0.67, 0.76]	
Xiao (2020)	Positive affect	elicitation	0.00 [−0.48, 0.48]	Single-acute intervention
	Positive affect	elicitation	−0.25 [−0.73, 0.24]	Long-term intervention
Zhou (2024b)	Negative affect	IAPS	−1.19 [−1.81, −0.57]	Single-acute intervention
	Negative affect	Cognitive Reappraisal	−1.22 [−1.84, −0.60]	
	Negative affect	Expressive Suppression	−1.22 [−1.84, −0.60]	
Nouchi et al. (2020)	Cognitive function	rST Middle-aged women	0.98 [0.44, 1.53]	Single-acute intervention
	Cognitive function	rSTOlder adult women	0.55 [0.03, 1.08]	
	Cognitive function	ST Middle-aged women	0.90 [0.36, 1.44]	
	Cognitive function	ST Older adult women	0.70 [0.18, 1.23]	
	Positive affect	POMS-SF2 Middle-aged women	1.27 [0.64, 1.89]	
	Positive affect	POMS-SF2 Older adult women	0.65 [0.06, 1.23]	
Yildirim et al. (2024)	Cognitive function	PASATLI	−0.19 [−0.75, 0.38]	Single-acute intervention
		MI	1.76 [1.12, 2.39]	
Luo (2022)	Sleep quality	PSQI	0.04 [−0.57, 0.66]	Long-term intervention
	Negative affect	VAS	0.32 [−0.30, 0.94]	
		SAI	−0.38 [−1.00, 0.24]	
Molina-Hidalgo et al. (2025)	Negative affect	BDI-II	−1.87 [−2.68, −1.06]	Long-term intervention
		/PSS		
		/POMS		
		/PANAS/SHS (Alcohol)		
	Negative affect	(Non-alcoholic)	−1.86 [−2.68, −1.05]	
Ni et al. (2024)	Cognitive function	2-back	0.92 [0.46, 1.38]	Long-term intervention
	Cognitive function	3-back	1.56 [1.05, 2.06]	
	Negative affect	HAMA	−0.45 [−0.89, −0.00]	
		HAMD	−0.46 [−0.91, −0.02]	
	Sleep quality	PSQI	−0.50 [−0.95, −0.06]	
Wang and Boros (2020)	Sleep quality	PSQI MI	−1.49 [−2.23, −0.75]	Long-term intervention
		PSQI HI	−1.70 [−2.46, −0.93]	
	Negative affect	STAI MI	−1.26 [−1.98, −0.54]	
	Negative affect	STAI HI	−1.18 [−1.89, −0.47]	
	Negative affect	SDS MI	−0.95 [−1.64, −0.26]	
	Negative affect	SDS HI	−1.23 [−1.94, −0.51]	
	Negative affect	SAI MI	−1.26 [−1.98, −0.54]	
	Negative affect	SAI HI	−1.18 [−1.89, −0.47]	
	Negative affect	TAI MI	−0.85 [−1.53, −0.17]	
	Negative affect	TAI HI	−0.90 [−1.59, −0.22]	
Zhou (2024b)	Cognitive function	MMSE	0.62 [0.03, 1.20]	Long-term intervention
	Cognitive function	MOCA	−0.05 [−0.50, 0.39]	
Xu et al. (2025)	Cognitive function	MMSE	−0.24 [−0.74, 0.27]	Long-term intervention
	Negative affect	HAMD-17	−0.53 [−0.90, −0.17]	
Cunha et al. (2025)	Cognitive function	MOCA good sleep	−0.85 [−1.30, −0.39]	Long-term intervention
	Cognitive function	MOCA poor sleep	−0.76 [−1.25, −0.27]	
	Sleep quality	PSQI	0.92 [0.42, 1.41]	
	Negative affect	BAI good sleep	0.43 [−0.01, 0.88]	
	Negative affect	BAI poor sleep	0.91 [0.41, 1.40]	
	Negative affect	PHQ-9 good sleep	0.30 [−0.14, 0.74]	
	Negative affect	PHQ-9 poor sleep	0.28 [−0.19, 0.76]	
Kargaran et al. (2021)	Cognitive function	MMSE	1.76 [0.75, 2.77]	Long-term intervention
	Sleep quality	PSQI	−0.63 [−1.64, 0.38]	
	Positive affect	SF-36	−0.05 [−0.90, 0.80]	
Wang and Boros (2020)	Sleep quality	PSQI	−0.11 [−0.88, 0.66]	Long-term intervention
You et al. (2023)	Cognitive function	Stroop congruent MI	0.60 [−0.12, 1.32]	Long-term intervention
	Cognitive function	Stroop congruent HI	0.00 [−0.68, 0.68]	
	Cognitive function	Stroop Incongruent MI	0.74 [0.01, 1.48]	
	Cognitive function	Stroop Incongruent HI	0.10 [−0.58, 0.78]	
Liu et al. (2025)	Cognitive function	SHMS	0.22 [−0.23, 0.68]	Long-term intervention
	Cognitive function	Visual memory test	0.60 [0.13, 1.06]	

LI is low intensity, MI is moderate intensity; HI is high intensity. PANAS is positive and negative affect schedule POMS is the Profile of Mood States, with subscales including T-A (Tension-Anxiety), D (Depression-Dejection), A-H (Anger-Hostility), V (Vigor-Activity), F (Fatigue-Inertia), C (Confusion-Bewilderment), S (Sadness), and TMD (Total Mood Disturbance) POMS-SF2 is the shortened form of the Profile of Mood States TAS is the Toronto Alexithymia Scale TAI is the Trait Anxiety Inventory STAI is the State–Trait Anxiety Inventory BDI-II is the Beck Depression Inventory-II PSS is the Perceived Stress Scale SHS is the Satisfaction with Life Scale HAMA is the Hamilton Anxiety Rating Scale; HAMD is the Hamilton Depression Rating Scale SDS is the Self-Rating Depression Scale BAI is the Beck Anxiety Inventory PHQ-9 is the Patient Health Questionnaire-9 VAS is the Visual Analog Scale PSQI is the Pittsburgh Sleep Quality Index MMSE is the Mini-Mental State Examination MoCA is the Montreal Cognitive Assessment 2-back and 3-back are working memory tasks in the n-back paradigm Stroop is the Stroop Color-Word Test, with conditions including congruent and incongruent trials PASAT is the Paced Auditory Serial Addition Test; rST is the reaction time Stroop Task ST is the Stroop Task SHMS is the Self-Rating Scale of Health Measurement System SF-36 is the Short Form-36 Health Survey IAPS is the International Affective Picture System and WLELS is the Well-Being Life Evaluation Scale.

#### 3.5.2 Acute aerobic exercise and negative mood

The leave-one-out sensitivity analysis showed that the pooled SMD ranged from −0.74 to −0.22 across the analyses. When the study by Wu and Zhou (2024) was excluded, the pooled SMD decreased to −0.33 [(95% CI: [−0.58, −0.09], *p* = 0.008)], and when Zhou (2024b) was removed, the SMD was −0.74 [(95% CI: [−1.29, −0.19], *p* = 0.008)], in both cases, the effect remained statistically significant. Similarly, excluding Liao (2023) or Qin (2023) resulted in SMDs of −0.38 [(95% CI: [−0.59, −0.18], *p* = 0.0003)] and −0.53 [(95% CI: [−0.81, −0.25], *p* = 0.0002)], respectively—both still significant. Notably, when both Zhou (2024b) and Liao (2023) were simultaneously removed, the pooled effect diminished to −0.22 [(95% CI: [−0.38, −0.06], *p* = 0.006)], indicating a reduced but still statistically significant effect (Table 3).

In all other exclusion scenarios, the effect sizes remained significant (*p* < 0.05), and the 95% CI did not cross zero (see Table 4). Heterogeneity varied across sensitivity analyses (*I^2^* from 0 to 84%), confirming the presence of substantial between-study heterogeneity. However, no single study or pairwise exclusion altered the direction of the effect (Table 5).

**Table 4:** Positive mood following acute aerobic exercise.

Study excluded (cumulative)	Overall effect (SMD)	95% CI	*I* ^2^	*Z*-value/*p*-value
None (original)	1.56	[0.63, 2.50]	94%	3.28/0.001
Nouchi et al. (2020)	1.79	[0.54, 3.04]	96%	2.81*/*0.005
Liao (2023)	0.33	[−0.07, 0.73]	66%	1.62/0.11
Qin (2023)	2.42	[1.00, 3.84]	96%	3.34/0.0008
Xiao (2020)	1.79	[0.72, 2.86]	95%	3.27/0.001
Nouchi et al. (2020); Liao (2023)	0.04	[−0.24, 0.31]	0%	0.25/0.80

**Table 5:** Negative mood following acute aerobic exercise.

Study excluded (cumulative)	Overall effect (SMD)	95% CI	*I* ^2^	*Z*-value/*p*-value
None (original)	−0.46	[−0.71, −0.21]	71%	3.58/0.0003
Wu and Zhou (2024)	−0.74	[−1.29, −0.19]	84%	2.64/0.008
Zhou (2024b)	−0.33	[−0.58, −0.09]	64%	2.64/0.008
Liao (2023)	−0.38	[−0.59, −0.18]	50%	3.66/0.0003
Qin (2023)	−0.53	[−0.81, −0.25]	73%	3.74/0.0002
Zhou (2024b); Liao (2023)	−0.22	[−0.38, −0.06]	0%	2.75/0.006

#### 3.5.3 Acute aerobic exercise on cognitive ability

The sensitivity analysis was conducted by sequentially removing one study at a time and re-running the meta-analysis. The results showed that upon the exclusion of any single study, the pooled effect size SMD fluctuated between −0.02 and 0.54. Specifically, when the study by Liao (2023) was excluded, the SMD increased significantly to 0.54 [(95% CI: 0.21 to 0.88), *p* = 0.001], changing from the original non-significant result (*p* = 0.26) to one of statistical significance (*p* < 0.05). Furthermore, when the studies by Liao (2023) and Yildirim et al. (2024) were simultaneously excluded, the SMD further increased to 0.50 [(95% CI: 0.26 to 0.75), *p* < 0.0001]. In the other leave-one-out analyses, while the effect size fluctuated slightly, it generally remained close to the significance level. Moreover, the 95% CI did not cross the null line, with the exception of the analysis excluding Nouchi et al. (2020), which yielded an effect size of −0.02 and a CI that included 0 (see Table 6).

**Table 6:** Cognitive performance following acute aerobic exercise.

Study excluded (cumulative)	Overall effect (SMD)	95% CI	*I* ^2^	*Z*-value/*p*-value
None (original)	0.24	[−0.17, 0.64]	85%	1.13/0.26
Yildirim et al. (2024)	0.14	[−0.26, 0.55]	82%	0.70/0.49
Nouchi et al. (2020)	−0.02	[−0.54, 0.51]	86%	0.07/0.95
Cui (2021)	0.23	[−0.21, 0.68]	86%	1.02/0.31
Liao (2023)	0.54	[0.21, 0.88]	72%	3.18/0.001
Qin (2023)	0.26	[−0.27, 0.79]	88%	0.95/0.34
Yildirim et al. (2024); Liao (2023)	0.50	[0.26, 0.75]	37%	3.99/0.0001

#### 3.5.4 Long-term aerobic exercise on negative mood

The sensitivity analysis showed that upon the exclusion of any single study, the SMD fluctuated between −0.86 and −0.25. When the study by Wang and Boros (2020) was excluded, the pooled effect size increased to −0.25 [(95% CI: −0.67, 0.17), *p* = 0.24]. In the remaining leave-one-out analyses, the effect size remained significant (*p* < 0.05), and all 95% CI did not cross the null line, suggesting that the direction of the overall effect was stable. Furthermore, heterogeneity fluctuated upon the exclusion of different studies (I^2^ range: 50–87%), indicating high heterogeneity among the studies, however, the exclusion of any single study did not alter the directionality of the result (as shown in Table 7).

**Table 7:** Negative mood following long-term aerobic exercise.

Study excluded (cumulative)	Overall effect (SMD)	95% CI	*I* ^2^	*Z*-value/*p*-value
None (original)	−0.58	[−0.91, −0.24]	85%	3.39/0.0007
Molina-Hidalgo et al. (2025)	−0.44	[−0.76, −0.12]	82%	2.70/0.007
Xu et al. (2025)	−0.58	[−0.95, −0.22]	85%	3.15/0.002
Cunha et al. (2025)	−0.86	[−1.13, −0.59]	65%	6.29/0.00001
Ni et al. (2024)	−0.60	[−0.99, −0.21]	86%	3.04/0.002
Wang and Boros (2020)	−0.25	[−0.67, 0.17]	87%	1.17/0.24
Luo (2022)	−0.64	[−1.01, −0.28]	86%	3.48/0.0005
Molina-Hidalgo et al. (2025); Cunha et al. (2025)	−0.72	[−0.95, −0.49]	50%	6.04 0.00001

#### 3.5.5 Long-term aerobic exercise on cognitive ability

The sensitivity analysis was conducted by sequentially removing one study at a time and re-running the meta-analysis. The results showed that upon the exclusion of any single study, the pooled effect size fluctuated between 0.17 and 0.54.

Specifically, when the study by Cunha et al. (2025) was excluded, the pooled effect size increased to 0.54 [(95% CI: 0.20, 0.87), *p* = 0.002]. This indicates that this particular study made the largest contribution to the overall effect. Conversely, when Ni et al. (2024) was excluded, the effect size decreased to 0.17 [(95% CI: −0.19, 0.52), *p* = 0.36]. In the remaining leave-one-out analyses, the effect size maintained a trend towards significance (*p* > 0.05, or in some cases still <0.05), and most 95% CI did not cross the null line, with the exception of Ni et al. (2024).

Furthermore, heterogeneity fluctuated upon the exclusion of different studies (*I*^2^ range: 76%–90%), indicating high heterogeneity among the studies, however, the exclusion of any single study did not alter the directionality of the result (as shown in Table 8).

**Table 8:** Cognitive performance following long-term aerobic exercise.

Study excluded (cumulative)	Overall effect (SMD)	95% CI	*I* ^2^	*Z*-value/*p*-value
None (original)	0.34	[−0.05, 0.73]	86%	1.70/0.09
Kargaran et al. (2021)	0.26	[−0.13, 0.65]	86%	1.29/0.20
Xu et al. (2025)	0.39	[−0.03, 0.81]	87%	1.82/0.07
Liu et al. (2025) (1)	0.33	[−0.14, 0.81]	88%	1.39/0.17
Cunha et al. (2025)	0.54	[0.20, 0.87]	76%	3.13/0.002
You et al. (2023) (5)	0.34	[−0.17, 0.85]	90%	1.32/0.19
Ni et al. (2024)	0.17	[−0.19, 0.52]	79%	0.91/0.36
Zhou (2024b)	0.36	[−0.10, 0.82]	88%	1.52/0.13

#### 3.5.6 Long-term aerobic exercise on sleep

The sensitivity analysis showed that upon the exclusion of any single study, the pooled effect size fluctuated between −0.83 and −0.01.

When the study by (Cunha et al., 2025) was excluded, the pooled effect size was significantly enhanced [(SMD = −0.83, 95% CI: [−1.46, −0.20], *p* = 0.01)], indicating that this study made a substantial contribution to the overall effect. Conversely, when the study by Wang and Boros (2020) was excluded, the pooled effect size approached null (SMD = −0.01, 95% CI: [−0.76, 0.75], *p* = 0.99) and was no longer statistically significant, suggesting that this study may have attenuated the overall effect. In the remaining leave-one-out analyses, although the effect sizes did not reach statistical significance (*p* > 0.05), their direction was consistent, and the 95% CI largely included or were close to the null line, with no change in directionality (as shown in Table 9).

**Table 9:** Sleep quality following long-term aerobic exercise.

Study excluded (cumulative)	Overall effect (SMD)	95% CI	*I* ^2^	*Z-*value/*p*-value
None (original)	−0.53	[−1.33, 0.27]	90%	1.30/0.19
Kargaran et al. (2021)	−0.52	[−1.42, 0.39]	92%	1.12/0.26
Cunha et al. (2025)	−0.83	[−1.46, −0.20]	95%	2.58/0.01
Ni et al. (2024)	−0.55	[−1.61, 0.51]	91%	1.01/0.31
Wang and Boros (2020)	−0.01	[−0.76, 0.75]	84%	0.01/0.99
Luo (2022)	−0.66	[−1.63, 0.32]	91%	1.32/0.19

## 4 Discussion

This meta-analysis of 19 randomized controlled trials evaluates the effects of aerobic exercise on cognition, mood, and sleep in healthy adults. Acute aerobic exercise significantly enhances positive mood and reduces negative mood, supporting its role as a rapid, non-pharmacological strategy for emotional regulation. Long-term aerobic exercise also significantly alleviates negative mood, but improvements in positive mood, cognitive performance, and sleep quality were not statistically significant. High heterogeneity across outcomes (*I*^2^ > 50%) suggests variability in intervention protocols, population characteristics, and measurement tools as key sources of inconsistency. Specifically, varying exercise intensities (low vs. moderate vs. high) and the mix of subjective versus objective measurement tools may be primary drivers of this heterogeneity. While Northey et al. (2018) reported significant cognitive benefits in older adults, our findings in healthy adults were non-significant—possibly due to age-related neuroplasticity differences or insufficient intervention intensity and cognitive task diversity. Notably, sensitivity analysis revealed that the pooled estimate for cognitive function became statistically significant when excluding Liao (2023). This suggests that the overall non-significant result may be driven by a few influential studies rather than a true absence of effect. Combined with the potential “ceiling effect” in healthy adults (where baseline cognitive performance is already high), we posit that the null findings for cognition may stem from both baseline limitations and study-specific variations.

Similarly, although Erickson emphasized cognitive gains from long-term exercise (Erickson et al., 2011), our observed trend did not reach significance, likely attenuated by heterogeneity. In contrast, our mood findings align with prior evidence that the immediate mood-enhancing effect of acute exercise echoes Bartholomew et al. (2005) conclusions, and the sustained reduction in negative mood supports Schuch et al. (2016) findings on exercise’s antidepressive potential, reinforcing the dual temporal model of mood regulation—acute exercise as emotional first-aid, long-term exercise for chronic mood management.

The observed effects may be mediated by multiple neurobiological pathways: (1) enhanced neuroplasticity via BDNF release in the hippocampus and prefrontal cortex (Erickson et al., 2011), supporting synaptic plasticity and neurogenesis (Cotman et al., 2007), (2) rapid upregulation of dopamine, serotonin, and endorphins during acute exercise, contributing to emotional reward and stress buffering (Basso and Suzuki, 2017; Meeusen and De Meirleir, 1995), (3) improved heart rate variability through autonomic nervous system rebalancing, promoting emotional stability and better sleep (Thayer et al., 2012; Michael et al., 2017), (4) long-term suppression of pro-inflammatory cytokines, potentially mediating mood and sleep benefits (Gleeson et al., 2011; Schuch et al., 2016), and (5) synchronization of circadian rhythms via exercise as a non-photic zeitgeber, stabilizing sleep–wake cycles (Youngstedt et al., 2019; Atkinson et al., 2007). These mechanisms form an integrated physiological network, though their interactions and population-specific weighting require further study using multimodal imaging and longitudinal designs.

In addition, despite the limitations of moderate-to-high heterogeneity, small sample sizes for some outcomes, and lack of standardized dosing, this study provides evidence that acute aerobic exercise is effective for rapid mood regulation, while long-term exercise supports emotional well-being. However, these findings are specific to healthy adult populations and should not be directly generalized to clinical groups without further evidence. The lack of significant effects on cognition and sleep may reflect insufficient power, suboptimal intervention parameters, or the aforementioned ceiling effect. Furthermore, the optimal dose–response relationship remains uncertain due to the lack of standardized protocols. Future research should establish standardized exercise prescriptions and unified assessment tools to enable dose–response modeling and advance toward precision exercise interventions. Nevertheless, current evidence supports integrating aerobic exercise—particularly acute sessions—into mental health promotion strategies for healthy populations.

## 5 Limitations

While this study highlights the benefits of exercise interventions, the interpretation of our findings is constrained by several methodological heterogeneities and limitations in evidence quality. First, significant clinical and methodological heterogeneity was observed across included studies regarding population characteristics, intervention protocols, and measurement tools. Specifically, the lack of consistency in exercise intensity served as a key driver of high heterogeneity. Furthermore, the use of diverse cognitive assessment tools and sleep scales across studies exacerbated the uncertainty in pooling effect sizes. Although a random-effects model was employed to account for some heterogeneity, residual variation may still compromise the precision and generalizability of the effect estimates.

Second, risk of bias assessment indicated that over 40% of the included studies exhibited a high risk of bias in the randomization process, coupled with a general lack of blinding for participants and outcome assessors. Given the highly subjective nature of mood and sleep indicators, the observed mood improvement effects should be interpreted with caution. Regarding publication bias, quantitative assessment was not feasible for most comparisons due to limited sample sizes (*n* < 10). However, visual asymmetry in funnel plots suggests potential publication bias, and the absence of negative or null results in published literature may lead to an overestimation of true effects.

Third, insufficient reporting of intervention adherence data, particularly in long-term interventions, introduces potential implementation bias. This makes it difficult to discern whether null findings in cognition and sleep stem from intervention inefficacy or adherence decay. Additionally, the generally high baseline cognitive levels in healthy adults suggest a potential “ceiling effect,” which may compress the room for improvement and contribute to the non-significant cognitive results.

Finally, limitations in statistical power and follow-up duration warrant attention. The insufficient sample size for long-term positive mood and sleep quality outcomes increases the risk of Type II errors. Crucially, most existing literature focuses on interventions within 12 weeks, with a severe lack of follow-up data beyond 6 months, preventing an effective evaluation of the durability and maintenance effects of exercise. In conclusion, future research should prioritize establishing standardized exercise prescription frameworks, optimizing randomization and blinding procedures, and standardizing adherence reporting. Large-scale, multi-center, long-term follow-up RCTs are recommended to solidify the evidence base for exercise interventions promoting mental health. Integrating wearable devices to dynamically monitor exercise load and physiological parameters will be key to advancing precision exercise prescriptions and elucidating dose–response relationships.

## 6 Practical implications and future research directions

The results of this study indicate that aerobic exercise holds clear clinical value and application potential in mental health promotion:

*Acute exercise as an emotional first-aid tool*: A single session of aerobic exercise can significantly boost positive mood and alleviate stress responses. It is recommended for use as an immediate mood regulation strategy in high-pressure situations.

Long-term exercise as a cornerstone intervention for mental health Regular aerobic exercise has been proven to effectively reduce anxiety and depression levels. It should be integrated into public health systems and clinical psychology intervention pathways as a non-pharmacological, low-cost, and high-adherence routine method for promoting mental health. The need to establish personalized exercise prescriptions Given that individuals respond differently to exercise interventions based on their goals, clinical practice should move toward developing goal-oriented exercise prescriptions to achieve precision intervention. To deepen the evidence base in exercise psychology and promote translational applications, subsequent research should focus on the following directions.

In summary, exercise is not only good medicine for physical health but also a prescription for mental health. The future lies in deepening our understanding of the mechanisms, standardizing protocols, and refining population-specific approaches to transform exercise intervention into a scalable, sustainable, and quantifiable public health action. It is recommended that education, healthcare, and sports authorities jointly develop international guidelines for exercise in mental health and incorporate scientific exercise prescriptions into health management processes in schools, corporations, and communities, thereby popularizing the concept and institutionalizing the practice of exercise as therapy.

## 7 Conclusion

Results from this meta-analysis indicate that acute aerobic exercise significantly improves positive mood and alleviates negative mood in healthy adults, long-term aerobic exercise also shows significant benefits in reducing anxiety and depressive symptoms, both supporting its role as an immediate, non-pharmacological strategy for emotional regulation. However, inconsistent improvements were observed for cognitive function or sleep quality, and no differences were found between exercise types or delivery formats. These effects may be dose-dependent, with longer and more structured interventions showing greater potential, though current evidence is limited by high heterogeneity, small sample sizes for some outcomes, and lack of standardized protocols. Further research is needed to determine the optimal type, dose, and duration of aerobic exercise, as well as the underlying mechanisms linking exercise to psychological benefits. Despite these limitations, the existing evidence is sufficient to justify the inclusion of aerobic exercise, as both acute and sustained intervention, into mental health promotion strategies for healthy populations.

## Data Availability

The original contributions presented in the study are included in the article/supplementary material, further inquiries can be directed to the corresponding author.
